# Supplementary material for: HARMONIES: A Hybrid Approach for Microbiome Networks Inference via Exploiting Sparsity
Source: Front Genet. 2020 Jun 3;11:445. doi: 10.3389/fgene.2020.00445 (PMC7283552; doi:10.3389/fgene.2020.00445)
Supplement: Supplementary file 1 [file Presentation_1.pdf]

Supplementary Material of HARMONIES: A Hybrid Approach for Microbiome Networks Inference via Exploiting Sparsity

1 MCMC ALGORITHM

We start by writing the likelihood for each sample $i, i = 1, \ldots, n$ as

$$f_{\text{ZINB}}(\boldsymbol{y}_{i\cdot}|\boldsymbol{\alpha}_{i\cdot}, \boldsymbol{\eta}_{i\cdot}, \boldsymbol{\phi}, s_i) = \prod_{j=1}^{p} f_{\text{ZINB}}(y_{ij}|\alpha_{ij}, \eta_{ij}, \phi_j, s_i),$$

where

$$\begin{aligned} & f_{\text{ZINB}}(y_{ij}|\alpha_{ij}, \eta_{ij}, \phi_j, s_i) \\ =& \mathrm{I}(y_{ij} = 0)^{\eta_{ij}} \left(\frac{\Gamma(y_{ij} + \phi_j)}{y_{ij}!\Gamma(\phi_j)} \left(\frac{\phi_j}{s_i\alpha_{ij} + \phi_j} \right)^{\phi_j} \left(\frac{s_i\alpha_{ij}}{s_i\alpha_{ij} + \phi_j} \right)^{y_{ij}} \right)^{1-\eta_{ij}}. \end{aligned}$$

Update of zero-inflation indicator η_{ij}: We update each $\eta_{ij}, i = 1, \ldots, n, j = 1, \ldots, p$ that corresponds to $y_{ij} = 0$ by sampling from the normalized version of the following conditional:

$$p(\eta_{ij}|\cdot) \propto f_{\text{ZINB}}(y_{ij}|\alpha_{ij}, \eta_{ij}, \phi_j, s_i) \cdot \text{Bern}(\eta_{ij}; \pi_i).$$

After the Metropolis-Hasting steps for all η_{ij}, we use a Gibbs sampler to update each $\pi_i, i = 1, \ldots, n$:

$$\pi_i|\cdot \sim \text{Be}(a_\pi + \sum_{j=1}^{p} \eta_{ij}, b_\pi + p - \sum_{j=1}^{p} \eta_{ij}).$$

Update of dispersion parameter ϕ_j: We update each $\phi_j, j = 1, \ldots, p$ by using a random walk Metropolis-Hastings algorithm. We first propose a new ${\phi_j}^*$ from $\text{Ga}({\phi_j}^2/\tau_\phi, \phi_j/\tau_\phi)$ and then accept the proposed value ${\phi_j}^*$ with probability $\min(1, m_{\text{MH}})$, where

$$m_{\text{MH}} = \frac{\prod_{i=1}^{n} f_{\text{ZINB}}(y_{ij}|\alpha_{ij}, \eta_{ij}, \phi_j, s_i)}{\prod_{i=1}^{n} f_{\text{ZINB}}(y_{ij}|\alpha_{ij}, \eta_{ij}, \phi_j, s_i)} \frac{\text{Ga}({\phi_j}^*; a_\phi, b_\phi)}{\text{Ga}(\phi_j; a_\phi, b_\phi)} \frac{J(\phi_j; {\phi_j}^*)}{J({\phi_j}^*; \phi_j)}.$$

Here we use $J(\cdot|\cdot)$ to denote the proposal probability distribution for the selected move. Note that the last term, which is the proposal density ratio, can be canceled out for this random walk Metropolis update.

Update of size factor s_i: We can rewrite Equation (2) in the main text, i.e.

$$\log s_i \sim \sum_{m=1}^{M} \psi_m \left[t_m \,\text{N}(\nu_m, \sigma_s^2) + (1 - t_m) \,\text{N}\left(-\frac{t_m \nu_m}{1 - t_m}, \sigma_s^2 \right) \right]$$

by introducing latent auxiliary variables to specify how each sample (in terms of $\log s_i$) is assigned to any of the inner and outer mixture components. More specifically, we can introduce an $n \times 1$ vector of assignment indicators $\boldsymbol{g}$, with $g_i = m$ indicating that $\log s_i$ is a sample from the m-th component of the outer mixture. The weight ψ_m determines the probability of each value $g_i = m$, with $m = 1, \ldots, M$. Similarly, we can consider an $n \times 1$ vector $\boldsymbol{\epsilon}$ of binary elements ϵ_i, where $\epsilon_i = 1$ indicates that, given $g_i = m$, $\log s_i$ is drawn from the first component of the inner mixture, i.e. $\mathrm{N}(\nu_m, \sigma_s^2)$ with probability t_m, and $\epsilon_i = 0$ indicates that $\log s_i$ is drawn from the second component of the inner mixture, i.e. $\mathrm{N}\left(-\frac{t_m \nu_m}{1-t_m}, \sigma_s^2\right)$, with probability $1 - t_m$. Thus, the Dirichlet process prior (DPP) model can be rewritten as

$$\log s_i | g_i, \epsilon_i, \boldsymbol{t}, \boldsymbol{\nu} \sim \mathrm{N}\left(\epsilon_i \nu_{g_i} + (1 - \epsilon_i)\frac{-t_{g_i}\nu_{g_i}}{1 - t_{g_i}}, \sigma_s^2\right),$$

where $\boldsymbol{t}$ and $\boldsymbol{\nu}$ denote the collections of t_m and ν_m, respectively. Therefore, the update of the size factor $s_i, i = 1, \ldots, n$ can proceed by using a random walk Metropolis-Hastings algorithm. We propose a new $\log s_i^*$ from $\mathrm{N}(\log s_i, \tau_s^2)$ and accept it with probability $\min(1, \mathrm{m_{MH}})$, where

$$\mathrm{m_{MH}} = \frac{\prod_{j=1}^{p} f_{\mathrm{ZINB}}(y_{ij}|\alpha_{ij}, \eta_{ij}, \phi_j, s_i^*)}{\prod_{j=1}^{p} f_{\mathrm{ZINB}}(y_{ij}|\alpha_{ij}, \eta_{ij}, \phi_j, s_i)} \frac{\mathrm{N}(\log s_i^*; \epsilon_i \nu_{g_i} + (1 - \epsilon_i)\frac{-t_{g_i}\nu_{g_i}}{1-t_{g_i}}, \sigma_s^2)}{\mathrm{N}(\log s_i; \epsilon_i \nu_{g_i} + (1 - \epsilon_i)\frac{-t_{g_i}\nu_{g_i}}{1-t_{g_i}}, \sigma_s^2)} \times \frac{J(\log s_i; \log s_i^*)}{J(\log s_i^*; \log s_i)}.$$

Note that the last term, which is the proposal density ratio, equals 1 for this random walk Metropolis update. Since $\boldsymbol{g}$, $\boldsymbol{\epsilon}$, $\boldsymbol{t}$, and $\boldsymbol{\nu}$ have conjugate full conditionals, we use Gibbs samplers to update them one after another:

- Gibbs sampler for updating $g_i, i = 1, \ldots, n$, by sampling from the normalized version of the following conditional:
$$p(g_i = m|\cdot) \propto \psi_m \mathrm{N}\left(\log s_i; \epsilon_i \nu_m + (1 - \epsilon_i)\frac{-t_m \nu_m}{1 - t_m}, \sigma_s^2\right).$$
- Gibbs sampler for updating $\epsilon_i, i = 1, \ldots, n$, by sampling from the normalized version of the following conditional:
$$p(\epsilon_i|\cdot) \propto \begin{cases} (1 - t_m)\mathrm{N}\left(\log s_i; -\frac{t_m \nu_m}{1-t_m}, \sigma_s^2\right) & \text{if } \epsilon_i = 0 \\ t_m \mathrm{N}\left(\log s_i; \nu_m, \sigma_s^2\right) & \text{if } \epsilon_i = 1 \end{cases}.$$
- Gibbs sampler for updating $t_m, m = 1, \ldots, M$:
$$t_m|\cdot \sim \mathrm{Be}(a_t + \sum_{i=1}^{n} \mathrm{I}(g_i = m)\mathrm{I}(\epsilon_i = 1), b_t + \sum_{i=1}^{n} \mathrm{I}(g_i = m)\mathrm{I}(\epsilon_i = 0)).$$
- Gibbs sampler for updating $\nu_m, m = 1, \ldots, M$:
$$\nu_m|\cdot \sim \mathrm{N}\left(\frac{c_m/\sigma_s^2}{e_m/\sigma_s^2 + 1/\tau_\nu^2}, \frac{1}{e_m/\sigma_s^2 + 1/\tau_\nu^2}\right),$$

where $c_m = \sum_{\{i:g_i=m,\epsilon_i=1\}} \log s_i - \frac{t_m}{1-t_m} \sum_{\{i:g_i=m,\epsilon_i=0\}} \log s_i$ and $e_m = \sum_{i=1}^n \mathrm{I}(g_i = m)\mathrm{I}(\epsilon_i = 1) + \sum_{\{i:g_i=m,\epsilon_i=0\}} \left(\frac{t_m}{1-t_m}\right)^2$.

- Gibbs sampler for updating $\psi_m, m = 1, \ldots, M$ by stick-breaking process:

$$\begin{aligned}
\psi_1 &= v_1, \\
\psi_2 &= (1 - v_1)v_2, \\
&\vdots \\
\psi_M &= (1 - v_1) \cdots (1 - v_{M-1})v_M,
\end{aligned}$$

where $v_m|\boldsymbol{\nu} \sim \text{Be}\left(a_m + \sum_{i=1}^n \mathrm{I}(g_i = m), b_m + \sum_{i=1}^n \mathrm{I}(g_i > m)\right)$.

For the sake of convenience, we have copied Equation (3) in the main text here,

$$p(\boldsymbol{\alpha}_{\cdot j}) = (nh_0 + 1)^{-\frac{1}{2}} \frac{\Gamma\left(a_0 + \frac{n}{2}\right)}{\Gamma(a_0)} \frac{b_0^{a_0}}{\left\{b_0 + \frac{1}{2}\left[\sum_{i=1}^n \log \alpha_{ij}^2 - \frac{\left(\sum_{i=1}^n \log \alpha_{ij}\right)^2}{n+\frac{1}{h_0}}\right]\right\}^{a_0+\frac{n}{2}}}.$$

Update of normalized abundance: We update each $\alpha_{ij}, i = 1, \ldots, n, j = 1, \ldots, p$ by using a Metropolis-Hastings random walk algorithm. We first propose a new $\alpha_{ij}{}^*$ from $\mathrm{N}(\alpha_{ij}, \tau_\alpha^2)$, and then accept the proposed value with probability $\min(1, \mathrm{m_{MH}})$, where

$$\mathrm{m_{MH}} = \frac{f_{\text{ZINB}}(\boldsymbol{y}_{i\cdot}|\boldsymbol{\alpha}_{i\cdot}{}^*, \cdot)}{f_{\text{ZINB}}(\boldsymbol{y}_{i\cdot}|\boldsymbol{\alpha}_{i\cdot}, \cdot)} \frac{p\left(\boldsymbol{\alpha}_{\cdot j}{}^*|\gamma_j\right)}{p\left(\boldsymbol{\alpha}_{\cdot j}|\gamma_j\right)} \frac{J\left(\alpha_{ij}; \alpha_{ij}{}^*\right)}{J\left(\alpha_{ij}{}^*; \alpha_{ij}\right)}.$$

Note that the last term, which is the proposal density ratio, equals 1 for this random walk Metropolis update.

SUPPLEMENT TO SECTION 3.2: ANALYSIS OF MICROBIOME DATA FROM COLORECTAL CANCER PATIENTS

We carried out a simple sensitivity analysis to evaluate the model performance to the choice of the filtering threshold. As discussed in Section 3.2, we filtered out the genera with more than 50% of nonzero counts across the samples. Here, we changed the threshold from 50% to 10%. The new threshold left 92 and 84 genera in the CRC and control group, respectively. Figure S2 shows the network inferred under this new setting.

Our first observation is that there were a large number of similarities between the networks. For instance, in the CRC group, the relatively stronger associations remained the same, such as positive associations between *Bacteroides* and *Alistipes*, *Barnesiella* and *Alistipes*, *Blautia* and *Dorea*, *Streptococcus* and *Haemophilus*. Though the new CRC network did not show any negative partial correlations (denoted by the red edges), the negative associations in the original network were relatively weak and might not be as stable as the other edges. Notably, the "positive triangle" among the three genera *Fusobacterium*, *Peptostreptococcus*, and *Parvimonas* was again confirmed here. In the control group, the strong associations can be still detected, such as the positive associations between *Alistipes* and *Parabacteroides*, *Alistipes* and *Bacteroides*, and negative associations between *Bacteroides* and *Dorea*. It is interesting to point out that the networks based on the new threshold tended to have fewer edges.

We also observed that increasing the number of genera in the networks could introduce novel associations. For example, genera *Pantoea* and *Escherichia* were dropped from the original CRC group network, yet they established a positive association in the new network. Similarly, genera *Pantoea*, *Escherichia*, *Abiotrophia*, *Oxalobacter*, and *Desulfovibrio* were of low abundance in the healthy controls and hence were not considered in the model before. However, they were connected in the new network. These results estimated from those highly sparse genera may hint further biological validations.

2 INFER THE NORMALIZED ABUNDANCES FOR MULTIPLE GROUPS

In practice, when there are two groups of subjects in a microbiome study (e.g., subjects with two distinct phenotypes), the sequencing data usually include measurements on the same taxonomic features for all the subjects. Then, if the abundance of a taxon j does not differ between two groups, we can improve the posterior influence of $\log \boldsymbol{\alpha}_{\cdot j}$ by merging two groups to increase the sample size. On the other hand, if the taxon is associated with subject's condition, i.e., a taxon that changes its abundance between two groups in the study, the inference of $\log \boldsymbol{\alpha}_{\cdot j}$ should rely on each subject group.

With the goal of borrowing information to improve the posterior inference for certain taxa, we combine the original count matrix from two different groups, to generate the count matrix $\boldsymbol{Y}_{n\times p}$. Here, the sample size is $n = n_1 + n_2$, with n_1, n_2 representing the number of subjects in the first and the second groups, respectively. Meanwhile, we let $\boldsymbol{z} = (z_1, \ldots, z_n)^T$ to allocate the n subjects into two groups, with $z_i = 1$ or 2 indicating the group label of subject i. In practice, if taxon j is irrelevant to the subject's phenotype, its abundances should not be differentiating between two groups. However, if taxon j is associated with the disease, its abundance could either increase or decrease from healthy subjects to patients. Therefore, we model the normalized abundance α_{ij} as following:

$$\log \alpha_{ij}|\gamma_j \sim \begin{cases} \mathrm{N}(\mu_{0j}, \sigma^2_{0j}) & \text{if } \gamma_j = 0 \\ \mathrm{N}(\mu_{1j}, \sigma^2_{1j}) & \text{if } \gamma_j = 1 \text{ and } z_i = 1 \\ \mathrm{N}(\mu_{2j}, \sigma^2_{2j}) & \text{if } \gamma_j = 1 \text{ and } z_i = 2 \end{cases}. \tag{S1}$$

Here, γ_j is a latent binary variable, with $\gamma_j = 1$ if taxon j is differentially abundant between two groups, and $\gamma_j = 0$ otherwise. For the taxa with $\gamma_j = 0$, we can borrow information between groups to increase the sample size in estimating the corresponding posterior of $\log \boldsymbol{\alpha}_{\cdot j}$. As an extension to Section 2.1 where we assume $\log \alpha_{ij} \sim \mathrm{N}(\mu_j, \sigma^2_j)$, the current model includes μ_{0j}, μ_{1j}, and μ_{2j} as the mean parameters for the normal mixture model. Again, we take the conjugate Bayesian approach and impose the following priors for the parameters in the normal mixture model: $\mu_{0j} \sim \mathrm{N}(0, h_0\sigma^2_0)$, $\sigma^2_{0j} \sim \mathrm{IG}(a_0, b_0)$, $\mu_{kj} \sim \mathrm{N}(0, h_k\sigma^2_k)$ and $\sigma^2_{kj} \sim \mathrm{IG}(a_k, b_k)$ for $k = 1, 2$.

The estimation of γ_j's determines the resulted normalized abundance matrix. Specifically, for taxon j with $\gamma_j = 0$, we can impute the zeros due to missing by the posterior mean of $\log \boldsymbol{\alpha}_{\cdot j}$ calculated using information from both groups. As an extension to Equation (3) in the main text , the posterior of $\alpha_{\cdot j}|\gamma_j$ is as following:

$$p(\boldsymbol{\alpha}_{\cdot j}|\gamma_j) = (2\pi)^{-\frac{n}{2}} \times \begin{cases} \prod_{k=1}^{K} (n_k h_k + 1)^{-\frac{1}{2}} \frac{\Gamma\left(a_k + \frac{n_k}{2}\right)}{\Gamma(a_k)} \frac{b_k^{a_k}}{\left\{b_k + \frac{1}{2}\left[\sum_{\{i:z_i=k\}} \log \alpha^2_{ij} - \frac{\left(\sum_{\{i:z_i=k\}} \log \alpha_{ij}\right)^2}{n_k + \frac{1}{h_k}}\right]\right\}^{a_k + \frac{n_k}{2}}} & \text{if } \gamma_j = 1 \\ (nh_0 + 1)^{-\frac{1}{2}} \frac{\Gamma\left(a_0 + \frac{n}{2}\right)}{\Gamma(a_0)} \frac{b_0^{a_0}}{\left\{b_0 + \frac{1}{2}\left[\sum_{i=1}^{n} \log \alpha^2_{ij} - \frac{\left(\sum_{i=1}^{n} \log \alpha_{ij}\right)^2}{n + \frac{1}{h_0}}\right]\right\}^{a_0 + \frac{n}{2}}} & \text{if } \gamma_j = 0 \end{cases}, \tag{S2}$$

Therefore, we can obtain the posterior mean of $\log \boldsymbol{\alpha}_{\cdot j}$ by averaging over the log-transformed MCMC samples of $\boldsymbol{\alpha}_{\cdot j}$ after burn-in.

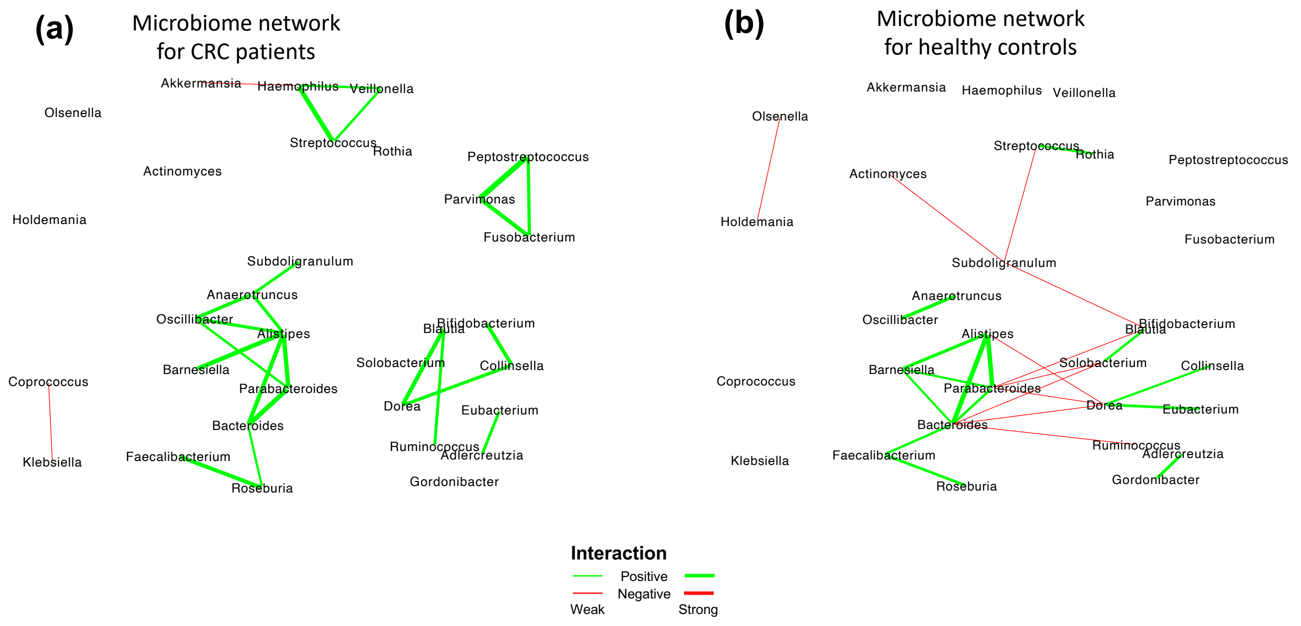

Figure S1. CRC case study: The estimated networks by HARMONIES for (a) CRC patients and (b) healthy controls. All nodes are labeled in their genus names.

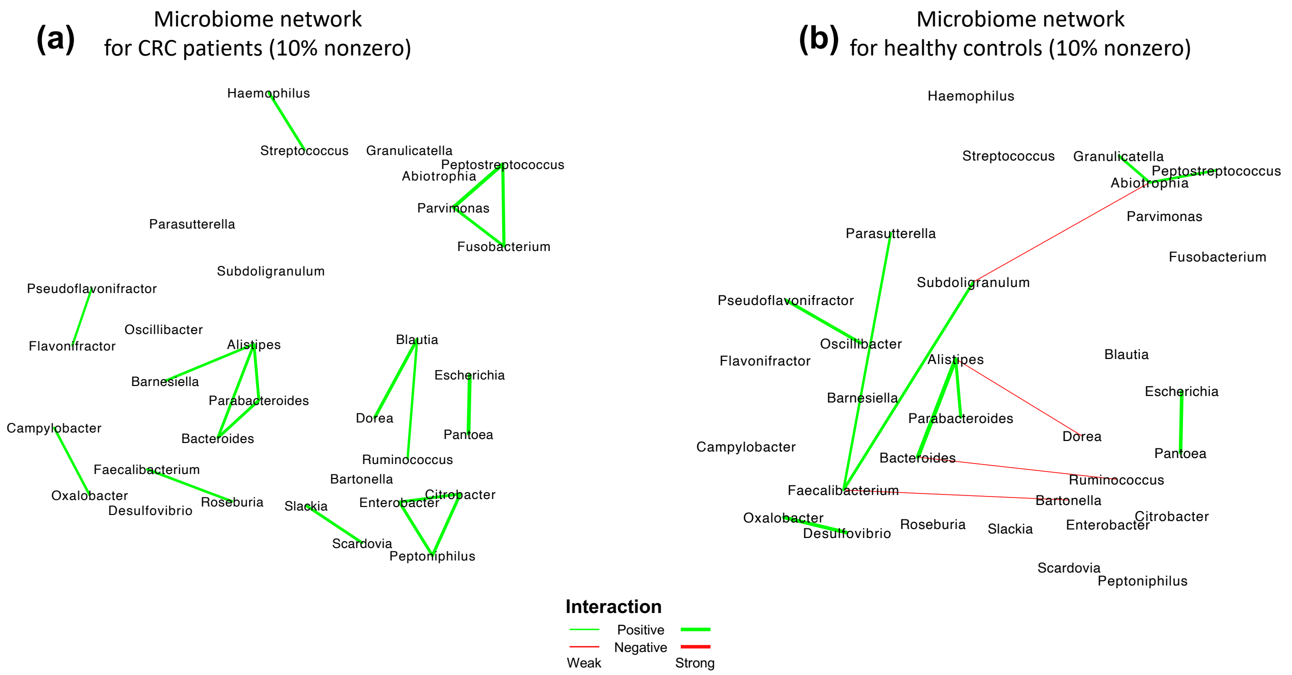

Figure S2. CRC case study: The estimated networks by HARMONIES for (a) CRC patients and (b) healthy controls. The nodes are genera that have at least 10% nonzero observations across the samples in each group.
